# Trends in asthma exacerbations in children before, during, and after the COVID-19 pandemic

**DOI:** 10.1017/S0950268825100800

**Published:** 2025-12-05

**Authors:** Liesa Stadhouders, Eline M. Hoogteijling, Liesbeth Duijts, Ankie Lebon

**Affiliations:** 1 Department of Pediatrics, Albert Schweitzer hospital, Dordrecht, The Netherlands; 2 Department of Pediatrics, division of Respiratory Medicine and Allergology, Erasmus MC, University Medical Center Rotterdam, Rotterdam, The Netherlands; 3 Department of Neonatal and Paediatric Intensive Care, division of Neonatology, Erasmus MC, University Medical Center Rotterdam, Rotterdam, The Netherlands

**Keywords:** allergy, asthma, bronchial hyperreactivity, COVID-19, paediatric asthma, PICU, salbutamol, viral wheezing, wheezing

## Abstract

Respiratory infections trigger asthma exacerbations. Despite being less severely affected by COVID-19 than adults, the subsequent lockdowns had a great impact on children. Previous studies showed a decrease in asthma exacerbations during the COVID-19 lockdowns, but findings from secondary care settings are scarce. We aimed to elucidate the trends in frequency and characteristics of asthma exacerbations in children presenting on an emergency department (ED) of a secondary care setting before, during, and after the COVID-19 pandemic. A retrospective analysis was conducted using data from ED visits between January 2018 and November 2022 for asthma exacerbations in children. The incidence of ED visits, hospital admissions, paediatric intensive care unit (PICU) admissions, administered medication, and demographic information were compared. A total of 1121 exacerbations were reported in 670 children, of whom 476 (42%) were admitted to hospital and 44 (3.9%) required PICU admission. We observed a decrease in ED visits for asthma exacerbations during the pandemic but an increased risk in hospital admissions and PICU transfers for exacerbations. This suggests a more severe course of exacerbations. Barriers to health care and lower viral exposure may contribute to this.

## Introduction

Upper respiratory infections are an important trigger for asthma and asthma-like exacerbations in children. Although children, regardless of their asthma status, are much less severely affected by COVID-19 than the adult population [1]. By social distancing and increased conscience about hygiene, not only the number of COVID-19 infections were limited, but all other respiratory and gastrointestinal infections in both adults and children also decreased in incidence during lockdown periods [2]. A lower viral exposure will lead to a reduction of asthma exacerbations [3]. However, hesitation to visit the emergency department may have led to a more severe course of the exacerbation, leading to an increased proportion of severe exacerbations and PICU admissions during the COVID-19 pandemic [4]. Moreover, limited possibilities for regular monitoring in outpatient clinics for both adults and children [5] and changes in medication adherence during COVID-19 pandemic may have affected the severity of asthma and asthma symptoms in children [6]. The COVID-19 pandemic also affected the reported incidence in new paediatric asthma diagnosis, which was greatly declined most probably due to decreased viral triggers and difficulty in healthcare access [7]. A potential increase in both asthma exacerbations and new asthma diagnoses could subsequently be expected when regulations were ended. In the Netherlands, most social restrictions for all residents were released in March 2022 [8], after which no new lockdown was needed.

We expect that due to a lower viral exposure a decrease in total asthma exacerbations during the pandemic occurred. After the pandemic, we expect an increase in asthma exacerbations and related increase in hospital and PICU admissions due to renewed viral exposure and the immunity debt build up during the lockdown period. Recent studies describe an increase in viral infections and severe asthma exacerbations after the COVID-19 pandemic [9–12]. To gain more sight into the course of exacerbations in our centre, we aimed to disentangle trends in frequency and characteristics of asthma exacerbations and admissions in children 1–18 years old before, during, and after the COVID-19 pandemic.

## Methods

### Patient identification

This retrospective cohort study used data collected from children aged 1–18 years with asthma and asthma-like exacerbations visiting the emergency department of the Albert Schweitzer hospital in Dordrecht, the Netherlands between January 2018 and November 2022. Patients were identified within the electronic patient files based on a registered Diagnosis Treatment Combination: ‘Asthma/bronchial hyperreactivity’. Data were extracted by the data management committee combined with manual data review and extraction. According to the Medical Research Involving Human Subjects Act (WMO), informed consent was not required. Incidence of emergency department visits, hospital admissions, and PICU admissions were visualized against a timeline. Duration of the pandemic defined as the first closure of primary schools at the beginning of the first lockdown 13 March 2020 until the opening of schools after the third and last lockdown on 10 January 2022 [8].

### Inclusion

Children visiting the emergency department multiple times during the same asthma exacerbation period (defined as 7 days) were counted as one. Admission was defined as all children for whom after the initial assessment was decided that prolonged inpatient treatment and monitoring was necessary and were thus moved to the paediatric ward. A severe exacerbation was defined as an exacerbation not responding to usual treatment and requiring intravenous salbutamol and transferal to the PICU. All children were treated according to the then current Dutch guidelines from the Dutch Association of Paediatrics. We collected data on the characteristics of all emergency department visits due to asthma exacerbations. We reported age, gender, medical history, admission history, and administered medication.

### Statistical analysis

Data are presented in frequencies and percentages for categorical variables. Relative risks were calculated using IBM SPSS Statistics 24 to compare risk of admission and medication used between times of presentation (before, during, and after the COVID-19 pandemic) and characteristics. The groups were compared in chronological order with the first group being the control and the next being the exposed group.

## Results

### Inclusion

**Figure 1.**
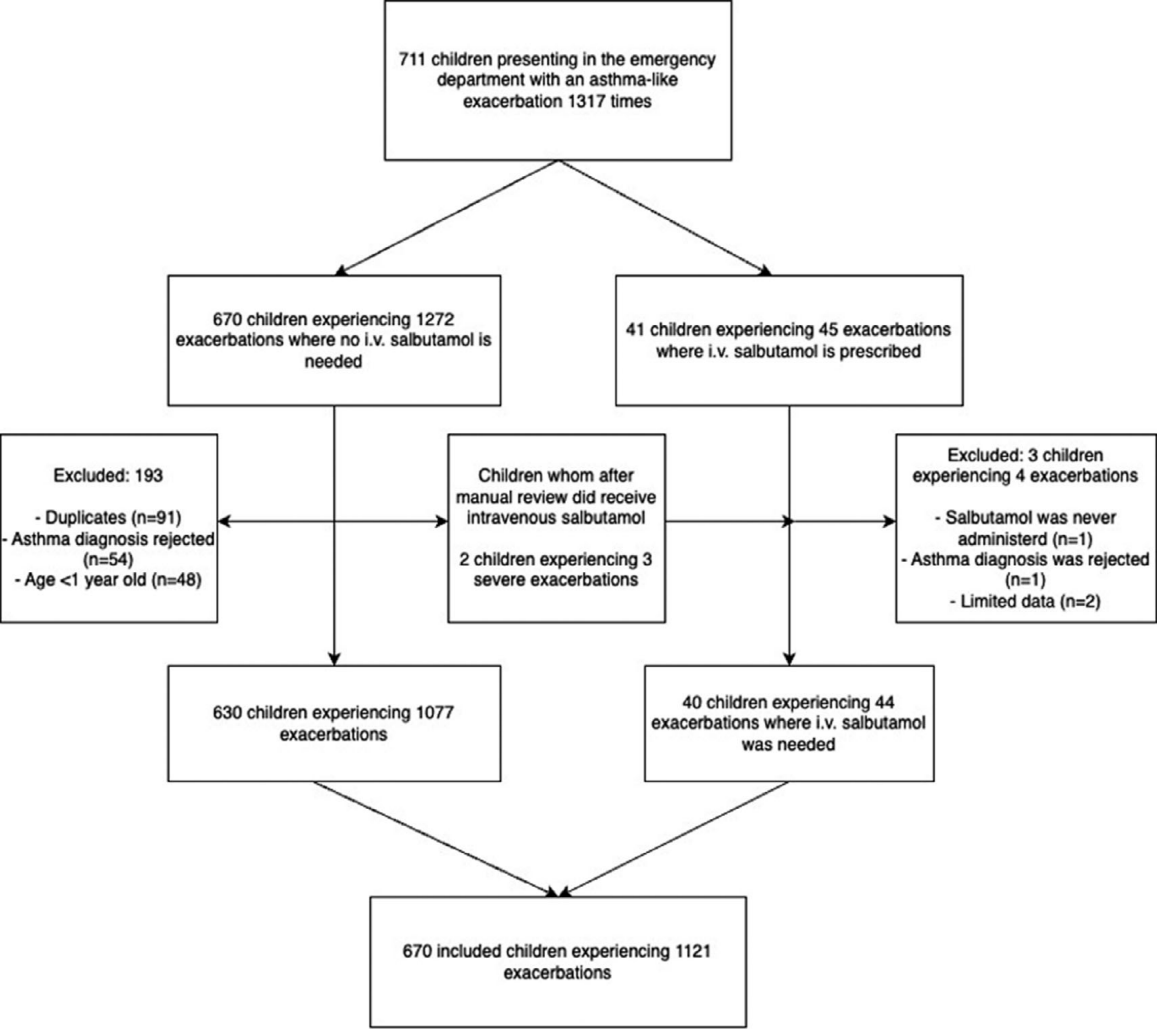
Flowchart of studypopulation.

**Figure 2.**
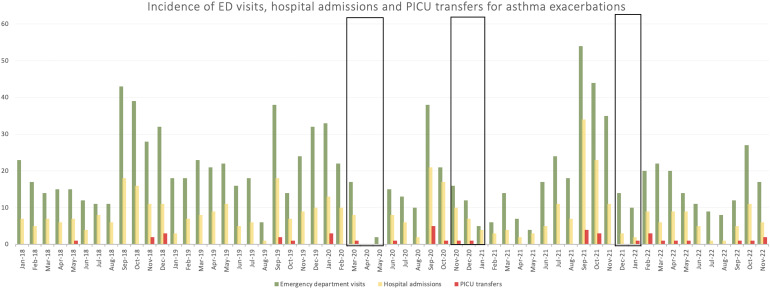
Frequency of emergency department (ED) visits, hospital admissions and PICU transfers for asthma exacerbations.

A total of 670 individual children have visited the emergency department due to an asthma exacerbation for a total of 1121 times in the selected timeframe. Inclusion process is visible in Figure 1. Transferral to the PICU occurred 44 times, in a total of 40 individual children. An overview of the frequencies per month for total exacerbations, hospital admissions, and PICU transfers is presented in Figure 2.

### Admission rates

Of all asthma exacerbations, 476 (42%) required admission to the general paediatric ward and 44 (3.9%) to the PICU. We observed a significant change in both the number of admissions and PICU transfers over the course of the COVID-19 pandemic. When comparing the individual time periods for admissions (Table 1), we observed an increase in admissions during the pandemic from 228/574 (39.7%) before to 184/379 (48.5%) during (RR 1.222 (1.058–1.412)), and a decrease after to 64/168 (38.1%) (RR 0.7847 (0.630–0.977)). We observed no significant difference in admission rates between before and after the pandemic (RR 0.959 (0.772–1.192)). For PICU admissions, we observed an increase when comparing both during and after pandemic of 19/379 (5.0%) and 12/168 (7.1%), respectively, with the period prior to the pandemic of 13/574 (2.3%) (RR 3.1538 (1.467–6.781) and 2.214 (1.106–4.428), respectively). As opposed to hospital admissions, no difference when comparing time periods during pandemic to after pandemic.

**Table 1.** Characteristics and frequencies of admissions and their relative risks for presenting with asthma exacerbations before, during, and after COVID-19 pandemic

	Total	Before pandemic	Before vs. during pandemic	During pandemic	During vs. after pandemic	After pandemic	Before vs. after pandemic
	*N* (%)	*N* (%)	RR (95% CI)	*N* (%)	RR (95% CI)	*N* (%)	RR (95% CI)
Total	1121	574		379		168	
Female sex	396 (35.3)	208 (36.2)	1.01 (0.85–1.19)	138 (36.4)	0.82 (0.63–1.07)	50 (29.8)	0.82 (0.64–1.06)
Age under 6 years	822 (73.3)	402 (70.0)	2.60 (2.27 to 2.98)*	295 (77.8)	1.16 (0.84–1.59)	125 (74.4)	0.85 (0.64–1.14)
History of asthma	631 (56.3)	352 (61.3)	0.79 (0.70–0.89)*	191 (50.4)	1.04 (0.87–1.24)	88 (52.4)	0.85 (0.73–1.00)
Previous admissions for asthma	474 (42.3)	266 (46.3)	0.79 (0.67–0.92)*	138 (36.4)	1.14 (0.92–1.43)	70 (41.7)	0.90 (0.74–1.10)
Total admissions	476 (42.5)	228 (39.7)	1.22 (1.06–1.41)*	184 (48.5)	0.785 (0.63–0.98)*	64 (38.1)	0.96 (0.77–1.19)
Total PICU transfers	44 (3.9)	13 (2.3)	2.21 (1.11–4.43)*	19 (5.0)	1.42 (0.71–2.87)	12 (7.1)	3.15 (1.47–6.78)*

History defined as previous presentation in the emergency department or outpatient clinic. Asthma exacerbations are presented as total numbers and their prevalences (%). Tests provided relative risks (RR) with their 95% confidence interval (CI). Significant results (*p* < 0.05) are indicated with *. PICU = paediatric intensive care unit.

### Risk characteristics

We observed that children 1–5 years of age had an increased risk of asthma exacerbations presenting at the emergency department during the pandemic compared to before with 402/574 (70.0%) and 295/379 (77.8%) cases, respectively (RR 2.598 (2.267–2.976)). In other words, young children seemed to be at a higher risk of having an asthma exacerbation during the pandemic than older children. Additionally, children with a history of asthma or that had previously been admitted to hospital both had a lower risk of asthma exacerbations during the pandemic than before with a decrease of 352/574 (61.3%) to 191/379 (50.4%) for a history of asthma and a decrease of 266/574 (46.3%) to 138/379 (36.4%) for children that have previously been admitted (0.7845 (0.695–0.886) and 0.786 (0.670–0.922), respectively). We observed no change in risk for asthma exacerbations after the pandemic ended and no change in risk when comparing presence of asthma exacerbations before and after pandemic for all characteristics. We observed no changes in use of prednisone and magnesium sulphate over the course of the pandemic, but we did for the use of intravenous salbutamol which was higher before and after the pandemic (Table 1). Since, at the time of the study, intravenous salbutamol was only given in the PICU setting, the statistics for PICU transfer are identical to those of intravenous salbutamol requirement.

## Discussion

During the pandemic, we observed a decrease in prevalence of asthma exacerbations in children compared to before the pandemic, but those that presented in the emergency room had an increased risk of admission to the hospital or PICU. This risk continued even after regulations were ended. This is in contrary to regular hospital admissions for asthma exacerbations for which the risk of hospital admission decreases to similar frequencies as before the pandemic when regulations are ended. In addition, we observed that the children with asthma exacerbations presenting at the emergency department during the pandemic were younger, and less often had a prior history of asthma or previous admissions. When looking at general trends, a couple of months after the ending of each lockdown period there seems to be a short spike in exacerbations and admissions, which seem in line with the seasonal highs, specifically at the end of summer 2021.

The decrease in asthma exacerbations during the pandemic may likely be attributed to reduced viral exposure due to social distancing measures and improved medication adherence, resulting in fewer asthma exacerbations [2, 3].

Prior studies have reported a decrease in both asthma exacerbations and emergency room visits [2–6, 9–12]. However, some of these describe an absolute decrease of both, while the proportion of admissions does increase. For example, Hurst et al. [12] describe a decrease in admission as a proportion of all healthcare encounters 97/3882 (2.5%) to 62/3307 (1.6%) translating to a relative risk of 0.745 (0.5436–1.0213). But looking at the proportion of admissions out of emergency department presentations results are in agreement with ours with 97/833 (11.6%) and 62/384 (16.1%) reflecting into an increased proportion of admissions and a relative risk of 1.333 (0.989–1.796) of children presenting in the emergency department with an asthma exacerbation. Although many of these studies do not describe the severity of exacerbations, the increased proportion of admissions could suggest that only more severe cases present to the emergency room, while milder exacerbations remain at home. While a reduction in asthma exacerbations during the pandemic initially seems positive, our study reveals an increase in the severity of exacerbations, leading to a relatively higher number of hospital admissions and a significant rise in PICU transfers, though the numbers are small. Several factors may contribute to this trend. For instance, it is possible that children with more severe or allergic asthma are continuing to experience exacerbations, but due to general discouragement to visit emergency facilities, present later with more severe symptoms resulting in a need for hospital admission or prednisone treatment. Alternatively, healthcare providers may have admitted children with asthma symptoms more often to monitor the course of disease, as there was a lack of knowledge on the effects of COVID-19 on children with asthma exacerbations at that time. However, this does not explain the steady increase in absolute numbers of severe asthma exacerbations requiring PICU transport for intravenous salbutamol during and after the lockdowns. Although our study did not collect data on treatment adherence among children on daily inhalation corticosteroids use, previous studies showed good or even improved adherence during the pandemic [5]. This is likely due to concerns about COVID-19’s impact on children with asthma or asthma symptoms. One hypothesis is the concept of ‘immune debt’ caused by reduced viral and bacterial exposure, leading to an increase in illness when regulations are lifted [13]. This aligns with our findings of a higher incidence of exacerbations and potentially increased severity shortly after the pandemic. A possible shift in dominant viral pathogens during and after the pandemic may also result in a shift in severe asthma exacerbations [2]. Another critical aspect influencing the rise in severe asthma exacerbations is the increased distance and more hesitation to visit healthcare facilities during the pandemic. During the peak pandemic periods, there was significant pressure on general practitioners and emergency rooms, resulting in higher barriers to care for non-COVID health issues. This delay in seeking care could contribute to the observed severity. Similarly, there may also be an increase in undertreated of newly onset asthma, where delays in referral and a higher number of telephone consultations may lead to delayed diagnosis and treatment [5, 7]. Children presenting at the emergency department during the COVID-19 pandemic were more likely to be under 6 years, have no previous history of asthma, and have not yet before been admitted for asthma exacerbations. This could also support the idea that barriers to both emergency and outpatient healthcare facilities might have been high, leading to more children suffering from undertreated asthma who consequently present in the emergency room with more severe asthma exacerbations.

The increasing rates of severe exacerbations is concerning. While the effects of a social distancing regulations, distance to health care, and immune debt might be part of observed associations, more factors may have had an influence on this.

### Limitations

This study gives a clear and compact overview of asthma exacerbations over the course of the pandemic and the trends over the different lockdown periods. However, several limitations must be acknowledged. First, our study is of a retrospective nature, which may have introduced inherent biases and limitations in data collection. Another limitation of this study is its single-centre design, which may restrict the generalizability of the findings. The heterogeneity among the study participants, stemming from diverse demographics and varying disease presentations, poses a challenge in drawing generalized conclusions. Additionally, we did not obtain any data on the patient characteristics and co-morbidities such as allergies, eczema, and current maintenance therapy. This could have provided more insight into the division between viral-induced and allergic-induced exacerbations.

## Conclusion

In conclusion, we observed a clear decrease in emergency department visits during the pandemic, especially during lockdown periods, followed by peaks shortly after restrictions were eased. However, we did not see a significant increase in asthma exacerbations after all regulations were lifted compared to pre-lockdown numbers when analysing a period of 10 months post-lockdown. Despite this, we observed a doubling of severe asthma exacerbations requiring PICU transfer with an incidence rising from 2.5% to 5% of all presenting exacerbations. This may have been a consequence of a combination of immune debt and under-treatment of asthma during lockdowns due to hesitancy to seek health care in an overwhelmed system. More research into the further course of incidence of severe asthma exacerbations in the years after the pandemic and more detailed characteristics of these children might help unravel the cause of this trend.

## Data Availability

Data are available upon reasonable request by contacting the corresponding author (Ankie Lebon – a.lebon@asz.nl).

## References

[1] Lee B, et al. (2022) Risk of serious COVID-19 outcomes among adults and children with moderate-to-severe asthma: A systematic review and meta-analysis. European Respiratory Review 31(166), 220066. 36323417 10.1183/16000617.0066-2022 PMC9724896

[2] Maison N, et al. (2022) The rising of old foes: Impact of lockdown periods on “non-SARS-CoV-2” viral respiratory and gastrointestinal infections. Infection 50(2), 519–524. 35076891 10.1007/s15010-022-01756-4 PMC8787179

[3] Kruizinga MD, et al. (2021) The impact of lockdown on pediatric ED visits and hospital admissions during the COVID-19 pandemic: A multicenter analysis and review of the literature. European Journal of Pediatrics 180(7), 2271–2279. 10.1007/s00431-021-04015-0. 33723971 PMC7959585

[4] Chelabi K, et al. (2023) The effect of the COVID-19 pandemic on pediatric asthma-related emergency department visits and hospital admissions in Montréal, Quebec: A retrospective cohort study. CMAJ Open 11(1), E152–E159. 10.9778/cmajo.20220072 PMC9933991 36787991

[5] Eguiluz-Gracia I, et al. (2021) Real-life impact of COVID-19 pandemic lockdown on the management of pediatric and adult asthma: A survey by the EAACI asthma section. Allergy 76(9), 2776–2784. 33772815 10.1111/all.14831 PMC8250685

[6] Feldman JM, et al. (2023) Reduced asthma morbidity during COVID-19 in minority children: Is medication adherence a reason? Journal of Asthma 60(3), 468–478. 10.1080/02770903.2022.2059510 PMC9532462 35341432

[7] Horton DB, et al. (2023) Childhood asthma diagnoses declined during the COVID-19 pandemic in the United States. Respiratory Research 24(1), 72. 36899362 10.1186/s12931-023-02377-7 PMC9999066

[8] Rijksoverheid (n.d.). *Coronavirus tijdlijn.* https://www.rijksoverheid.nl/onderwerpen/coronavirus-tijdlijn (accessed October 2025).

[9] Siu KK, et al. (2025). Population-based study on hospital admissions for pediatric status asthmaticus: From before to after the COVID-19 pandemic. Frontiers in Pediatrics, 13, 1534770. 10.3389/fped.2025.1534770. 40416442 PMC12098421

[10] Murata Y, et al. (2025) Impact of respiratory viral infections on pediatric inpatients with a respiratory disease after COVID-19 restrictions. Pediatrics International 67(1), e70138. 10.1111/ped.70138. 40598968

[11] Lukac CD, et al. (2025). Hospitalizations for all-cause pediatric acute respiratory diseases in Alberta, Canada, before, during, and after the COVID-19 pandemic. Lancet Regional Health – Americas, 44, 101024. 10.1016/j.lana.2025.101024. 40260184 PMC12010394

[12] Hurst JH, et al. (2021) Reduced pediatric urgent asthma utilization and exacerbations during the COVID-19 pandemic. Pediatric Pulmonology 56(10), 3166–3173. 34289526 10.1002/ppul.25578 PMC8441648

[13] Guadalupe-Fernández V, et al. (2024). Investigating epidemiological distribution of respiratory pathogens following COVID-19 de-escalation in Catalonia, September 2016–June 2021. PLoS One 19(2), e0285892. 38335176 10.1371/journal.pone.0285892 PMC10857536

